# HDAC8 drives spindle organization during meiotic maturation of porcine oocytes

**DOI:** 10.1111/cpr.13119

**Published:** 2021-08-25

**Authors:** Ying Chen, Chen Pan, Yajuan Lu, Yilong Miao, Bo Xiong

**Affiliations:** ^1^ College of Animal Science and Technology Nanjing Agricultural University Nanjing China; ^2^ State Key Laboratory for Conservation and Utilization of Subtropical Agro‐Bioresources Guangxi University Nanning China

**Keywords:** HDAC8, oocyte maturation, spindle assembly, γ‐tubulin

## Abstract

**Objectives:**

Histone deacetylase 8 (HDAC8) is one of the class I HDAC family proteins, which participates in the neuronal disorders, parasitic/viral infections, tumorigenesis and many other biological processes. However, its potential function during female germ cell development has not yet been fully understood.

**Materials and methods:**

HDAC8‐targeting siRNA was microinjected into GV oocytes to deplete HDAC8. PCI‐34051 was used to inhibit the enzyme activity of HDAC8. Immunostaining, immunoblotting and fluorescence intensity quantification were applied to assess the effects of HDAC8 depletion or inhibition on the oocyte meiotic maturation, spindle/chromosome structure, γ‐tubulin dynamics and acetylation level of α‐tubulin.

**Results:**

We observed that HDAC8 was localized in the nucleus at GV stage and then translocated to the spindle apparatus from GVBD to M II stages in porcine oocytes. Depletion of HDAC8 led to the oocyte meiotic failure by showing the reduced polar body extrusion rate. In addition, depletion of HDAC8 resulted in aberrant spindle morphologies and misaligned chromosomes due to the defective recruitment of γ‐tubulin to the spindle poles. Notably, these meiotic defects were photocopied by inhibition of HDAC8 activity using its specific inhibitor PCI‐34051. However, inhibition of HDAC8 did not affect microtubule stability as assessed by the acetylation level of α‐tubulin.

**Conclusions:**

Collectively, our findings demonstrate that HDAC8 acts as a regulator of spindle assembly during porcine oocyte meiotic maturation.

## Graphical abstract

Our findings document that HDAC8 locates to the spindle apparatus in porcine oocytes to take part in the spindle assembly via recruitment of γ‐tubulin to the spindle poles, and hence driving the oocyte meiotic maturation. This function of HDAC8 relies on its enzyme activity and is conserved among species in mammals.

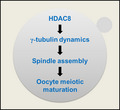

## 1 INTRODUCTION

The acetylation and deacetylation of histone are associated with multiple biological events via altering gene expression.^1^, ^2^, ^3^, ^4^, ^5^, ^6^, ^7^, ^8^ Histone deacetylases (HDACs) are a family of enzymes, which exert multifunctional, complicated functions in vivo, including promotion of DNA damage repair, regulation of cytoskeletal function, control of heart growth and modulation of thymocyte development through deacetylation of nuclear histone proteins and various non‐histone proteins.^9^, ^10^, ^11^, ^12^ Based on sequence homology, histone deacetylase 8 (HDAC8) is one of the class I HDAC enzymes, which plays important roles in parasitic/viral infections, tumorigenesis, neuronal disorders, smooth muscle contraction, skull morphogenesis, telomere protection and cohesin dynamics.^12^, ^13^, ^14^, ^15^, ^16^, ^17^

HDAC8 has been shown to target a non‐histone protein, the SMC3 subunit of cohesin.^17^ HDAC8‐mediated deacetylation of SMC3 is necessary for the cohesin complex recycle for use in the next cell cycle.^17^ Therefore, mutations of HDAC8 result in the Cornelia de Lange syndrome (CdLS), a dominantly inherited congenital malformation disorder.^17^ In addition, knockdown of HDAC8 by RNAi suppresses the growth of human lung, colon and cervical cancer cell lines, indicating the importance of HDAC8 for tumour cell proliferation.^18^ It has been also reported that HDAC8 promotes the long‐term hematopoietic stem cell maintenance and cell survival by modulating the activity of p53.^19^ In our previous study, HDAC8 has been found to locate at the spindle poles to maintain the spindle structure and chromosome alignment in mouse oocytes, suggesting that HDAC8‐mediated deacetylation is necessary for the faithful segregation of chromosomes and generation of euploid oocytes.^20^ The accurate segregation of genetic information between daughter cells are the most fundamental processes of life and requires a molecular machine built primarily of microtubules that form a specialized bipolar‐shaped spindle to drive the congression of chromosomes at metaphase I (M I) stage and the segregation of chromosomes at anaphase I (A I) stage in cells.^21^, ^22^, ^23^, ^24^ Thus, the precise spatiotemporal coordination of spindle assembly and chromosome alignment is essential for the orderly progression of oocyte maturation.^25^ Any mistake during this process is prone to chromosome segregation errors that would result in the aneuploid eggs.^26^ Whether the specific role of HDAC8 during meiotic spindle assembly in mouse oocytes is conserved among different species is still unknown.

In the present study, we explored the exact role of HDAC8 in porcine oocytes because of the developmental and maturational similarity between porcine and human oocytes. We found that HDAC8 co‐localized with spindle apparatus during porcine oocyte meiosis. Depletion of HDAC8 by RNAi led to impaired meiotic progression, spindle/chromosome structure and γ‐tubulin dynamics in porcine oocytes. These meiotic defects were dependent on the enzyme activity of HDAC8.

## 2 MATERIALS AND METHODS

### 2.1 Collection of porcine oocytes

Abattoir‐derived porcine ovaries were transported to the laboratory within 2 hours in a physiological saline containing penicillin G/streptomycin sulphate. Cumulus‐oocyte complexes (COCs) were isolated from the follicles (3‐6 mm in diameter) using a disposable syringe with a 20‐gauge needle.

### 2.2 In vitro maturation of porcine oocytes

Oocytes with a compact cumulus cells were used for in vitro maturation (IVM) in TCM‐199 (Thermo Fisher Scientific) supplemented with 10 ng/mL EGF, 5 μg/mL insulin, 0.2 mmol/L pyruvate, 0.6 mmol/L cysteine, 10% porcine follicular fluid, 10 IU/mL of each eCG and hCG and 25 μg/mL kanamycin. 20‐30 germinal vesicle (GV) oocytes were cultured for 26‐28 hours to metaphase I stage and for 44‐48 hours to metaphase II stage in 100 μL TCM‐199 covered with mineral oil at 38.5°C, 5% CO_2_.

### 2.3 cRNA construct and in vitro transcription

Wild‐type HDAC8 cDNA was sub‐cloned into pcDNA3.1/RFP vector. Capped cRNA was synthesized from linearized plasmid using T7 mMessage mMachine kit (Thermo Fisher Scientific) and purified with MEGAclear kit (Thermo Fisher Scientific). Typically, 10‐12 pL of 0.5‐1.0 μg/μL cRNA was injected into oocytes and then trained normally for further study.

### 2.4 SiRNA knockdown of HDAC8

Knockdown of HDAC8 was achieved through microinjection of porcine oocytes with 5‐10 pL of non‐targeting or HDAC8‐targeting siRNA, which were obtained from Genepharma, Shanghai, China. 25 μmol/L siRNA was used as working concentration. The sequences of HDAC8 siRNA: 5′‐GGUGUACAUAGCCUUUAAUTT‐3′. To facilitate the degradation of mRNA by siRNA, microinjected oocytes were arrested at GV stage in maturation medium containing 1 mmol/L dibutyryl‐cyclic‐AMP (dbcAMP) for 24 hours and then transferred to dbcAMP‐free maturation medium to resume the meiosis for further experiments.

### 2.5 Inhibitor treatment

PCI‐34051 was obtained from Santa Cruz Biotechnology and dissolved in DMSO to 100 mmol/L for a stock solution, which was further diluted with culture medium to a working concentration of 25 and 50 μmol/L respectively. The solvent was not diluted more than 0.1% of the maturation medium.

### 2.6 Immunofluorescence staining

Denuded oocytes were fixed in 4% paraformaldehyde/PBS (fixation solution) for 30 minutes and then transferred to 1% Triton X‐100 (permeabilization solution) for 8 hours. After washing in PBST, oocytes were incubated with 3% BSA/PBS (blocking solution) for 1 hour at room temperature (RT), followed by incubation with rabbit anti‐HDAC8 antibody (1:50; ab187139; Abcam), mouse anti‐α‐tubulin‐FITC antibody (1:200; F2168; Sigma‐Aldrich), rabbit anti‐γ‐tubulin antibody (1:50; ab179503; Abcam), sheep anti‐BubR1 antibody (1:50; ab28193; Abcam) or mouse anti‐acetylated‐α‐tubulin (Lys 40) antibody (1:50; T7451; Sigma‐Aldrich) at 4°C overnight. After three times of washes in PBST, oocytes were incubated with corresponding secondary antibodies for 1 hour and counterstained with 10 µg/mL propidium iodide (PI) or Hoechst 33342 for 10 minutes at RT. Finally, oocytes were mounted on the glass slides and imaged by the laser confocal microscope (LSM 900 META, Zeiss).

### 2.7 Immunoblotting analysis

Porcine oocytes were lysed in 4 × NuPAGE™ LDS sample buffer (Thermo Fisher Scientific) supplemented with protease inhibitor to attain total protein. Proteins were separated on 10% NUPAGE™ Bis‐Tris gels and afterwards transferred to polyvinylidene difluoride (PVDF) membranes. The blots were blocked with Tris‐buffered saline Tween 20 (TBST), supplemented with 5% low fat dry milk for 1 hour at RT and then incubated with the primary antibodies (anti‐HDAC8, 1:500; Abcam; anti‐acetylated‐α‐tubulin, 1:1000, Sigma‐Aldrich; anti‐γ‐tubulin, 1:500, Abcam; anti‐GAPDH, 1:1000, Cell Signaling Technology) at 4°C overnight. After washing three times in TBST, the blots were incubated with HRP‐conjugated secondary antibodies for 1 hour at RT. Lastly, protein bands were detected with ECL Plus (Thermo Fisher Scientific) using Tanon‐3900 Imaging System (Tanon, Shanghai, China).

### 2.8 Statistical analysis

All percentages or values from at least three independent replicates were expressed as mean ± SEM or mean ± SD, and the number of oocytes observed was labelled in parentheses as (n). Data were analysed by paired‐samples *t* test, provided by GraphPad Prism 8 statistical software. The level of significance was accepted as *P* < .05.

## 3 RESULTS

### 3.1 Subcellular localization and protein expression of HDAC8 in porcine oocytes

**FIGURE 1:**
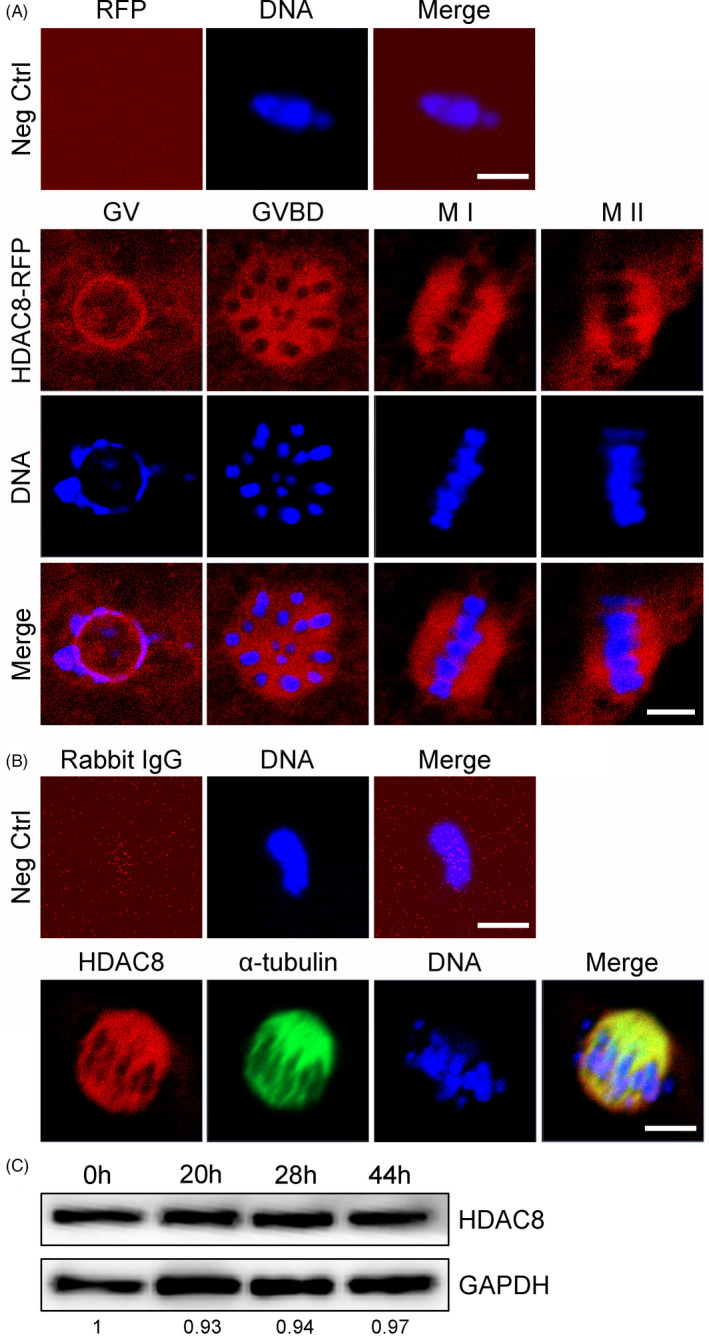
Subcellular localization and protein expression patterns of HDAC8 during meiotic maturation of porcine oocytes. (A) Localization of HDAC8‐RFP in porcine oocytes. GV oocytes were microinjected with RFP or HDAC8‐RFP cRNA and then in vitro cultured to different developmental stages for confocal imaging. Scale bar, 5 μm. (B) Co‐localization of HDAC8 with the spindle apparatus. M I oocytes were immunostained for HDAC8 and α‐tubulin, followed by DNA staining with Hoechst. Scale bar, 5 μm. (C) Protein levels of HDAC8 during porcine oocyte meiosis. Oocytes collected from different developmental stages were immunoblotted for HDAC8 and GAPDH respectively

We firstly microinjected HDAC8‐RFP cRNA into porcine oocytes to observe the subcellular localization of HDAC8‐RFP during oocyte meiotic maturation by confocal microscopy. As shown in Figure 1A, HDAC8‐RFP localized in the nucleus at GV stage and then translocated to the spindle apparatus from GVBD to M II stages in porcine oocytes (Figure 1A). The spindle localization of exogenous HDAC8 was confirmed by colocalization of endogenous HDAC8 with α‐tubulin in M I oocytes by antibody staining (Figure 1B). We then assessed the protein expression of HDAC8 at different developmental stages throughout the whole meiotic progression in porcine oocytes by immunoblotting analysis. The results indicated that protein levels of HDAC8 remained constant during the progression of oocyte maturation (Figure 1C). Thus, these observations suggest that HDAC8 might have functions related to the spindle dynamics during porcine oocyte meiotic maturation.

### 3.2 Depletion of HDAC8 impairs the normal progression of porcine oocyte meiosis

**FIGURE 2:**
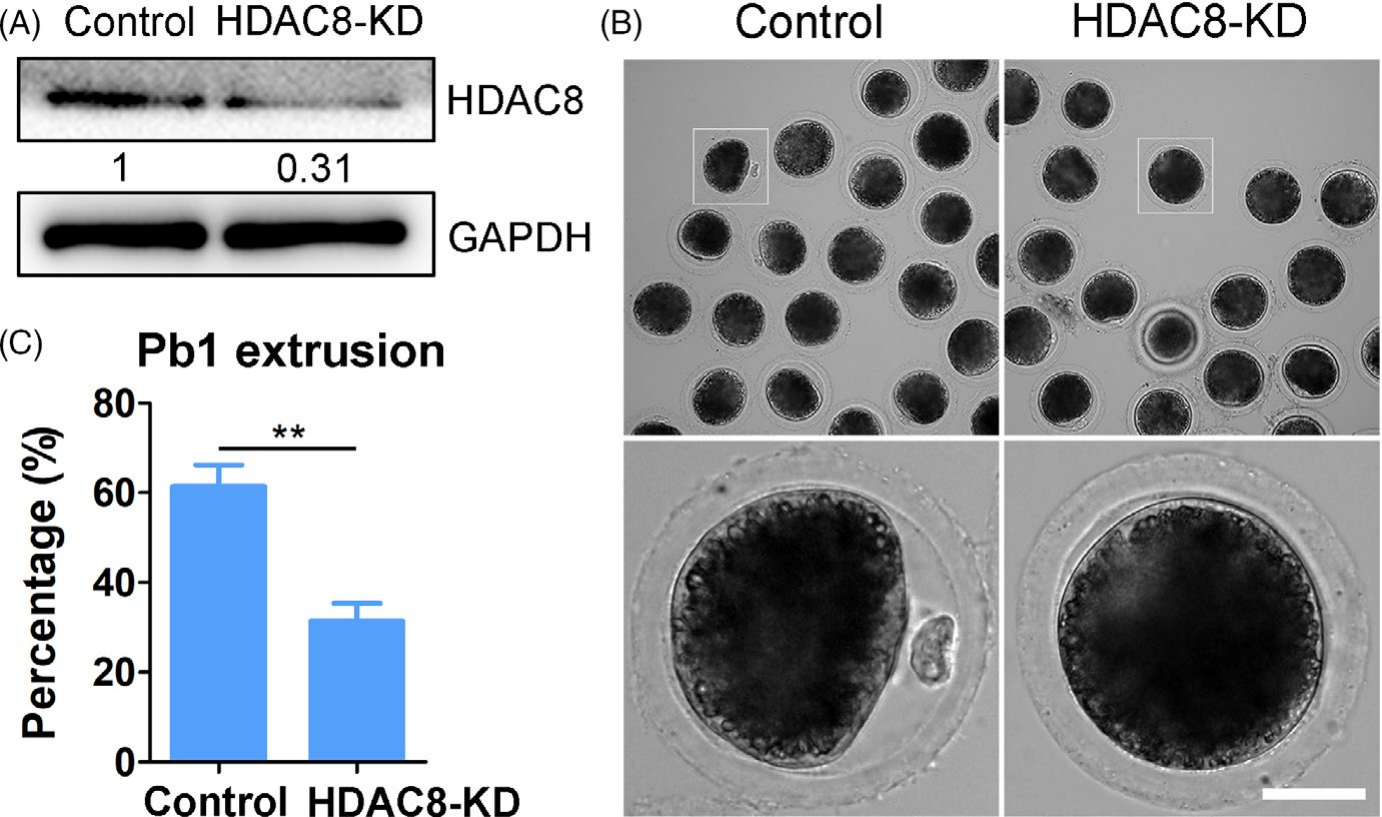
Effect of HDAC8 depletion on the meiotic progression in porcine oocytes. (A) Knockdown efficiency of RNAi‐mediated HDAC8 depletion. Protein levels of HDAC8 and GAPDH were assessed by immunoblotting analysis in control and HDAC8‐KD (siRNA‐injected) oocytes. (B) Representative images of oocytes after in vitro maturation in control and HDAC8‐KD groups. Scale bar, 30 μm. (C) The PBE rate was quantified in control and HDAC8‐KD oocytes. Data were presented as mean percentage (mean ± SEM) of at least three independent experiments. **P < .01

To investigate the specific role of HDAC8 in porcine oocytes, we applied RNAi‐mediated loss of function method to monitor the extrusion of the first polar body, the most critical indicator for oocyte maturation. GV oocytes were microinjected with porcine HDAC8‐targeting siRNA and then incubated in the medium containing dbcAMP for 24 hours to completely silence HDAC8, followed by in vitro maturation for 44 hours. Immunoblotting analysis revealed that HDAC8 protein level was significantly reduced after knockdown compared with the control (Figure 2A), validating the effectiveness of knockdown. We next evaluated the effect of HDAC8 depletion on the porcine oocyte meiotic maturation. We found that the rate of polar body extrusion (PBE) was remarkably decreased in HDAC8‐depleted oocytes compared with the controls (31.4 ± 3.9%, n = 69 vs 61.4 ± 4.8%, n = 73, *P* <.01; Figure 2B,C), implying that the normal meiotic maturation of porcine oocytes requires HDAC8.

### 3.3 Depletion of HDAC8 compromises the spindle assembly and chromosome alignment in porcine oocytes

**FIGURE 3:**
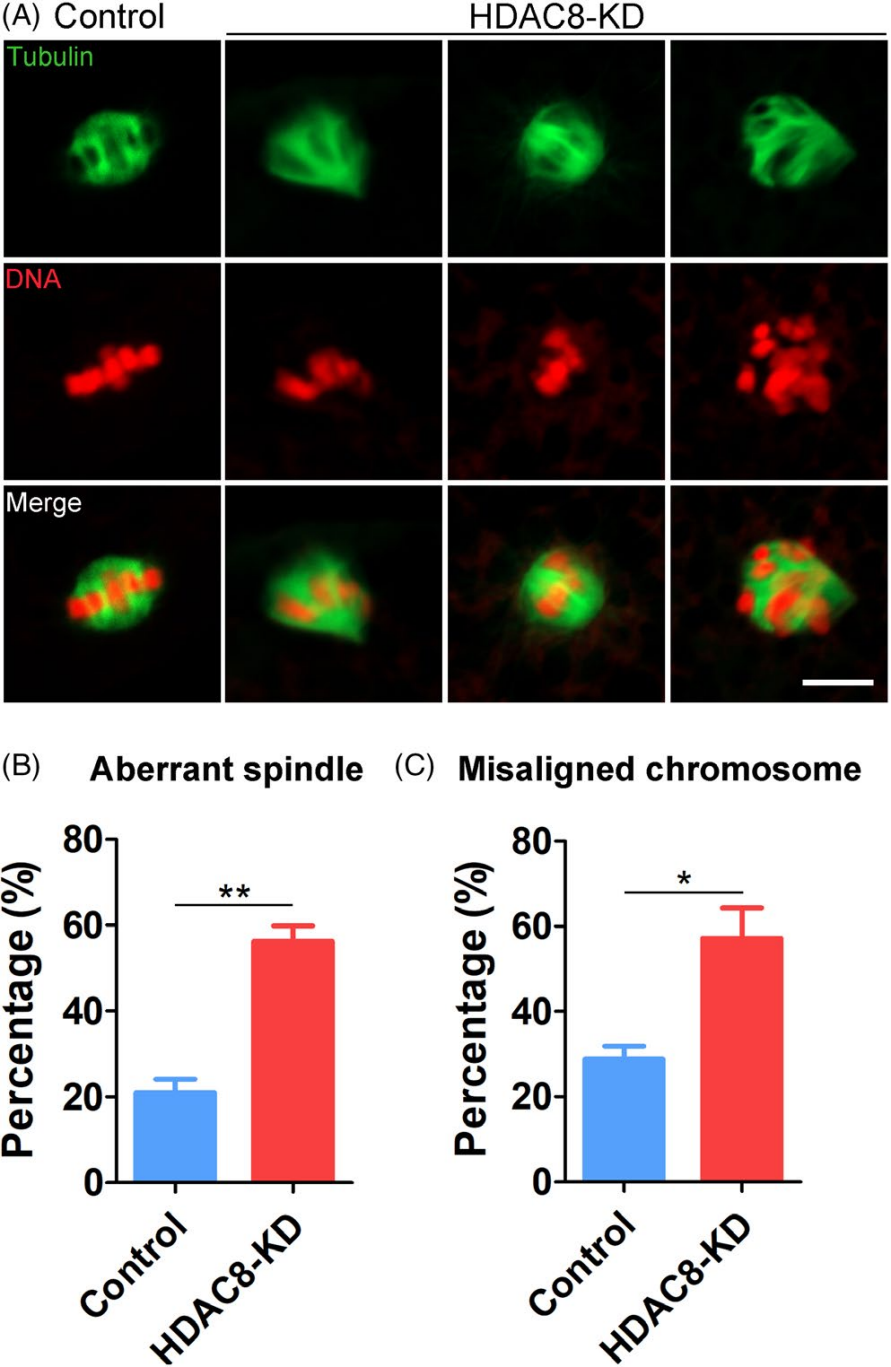
Effect of HDAC8 depletion on the spindle/chromosome structure in porcine oocytes. (A) Representative images of spindle morphology and chromosome alignment in control and HDAC8‐KD oocytes. M I oocytes were immunostained with anti‐α‐tubulin‐FITC antibody and counterstained with propidium iodide (PI). Scale bar, 5 μm. (B) The proportion of aberrant spindles was quantified in control and HDAC8‐KD oocytes. (C) The proportion of misaligned chromosomes was quantified in control and HDAC8‐KD oocytes. Data of (B) and (C) were presented as mean percentage (mean ± SEM) of at least three independent experiments. *P < .05, **P < .01

Given that HDAC8 localizes to the spindle and spindle/chromosome structure is a critical meiotic apparatus for oocyte maturation, we evaluated its organization in HDAC8‐depleted oocytes by immunofluorescence. To this end, we stained M I oocytes with α‐tubulin and counterstained chromosome with propidium iodide (PI). The imaging result showed that a typical bipolar spindle with a set of well‐aligned chromosomes at the equator was present in control oocytes (Figure 3A). However, an increased frequency of aberrant spindle (56.2 ± 3.6%, n = 46 vs 21.0 ± 3.0%, n = 45, *P* < .01; Figure 3B,C) with misaligned chromosomes (57.1 ± 7.1%, n = 46 vs 28.8 ± 3.0%, n = 45, *P* < .05; Figure 3B,C) was observed in HDAC8‐depleted oocytes. Therefore, these data indicate that HDAC8 might participate in spindle assembly to drive the porcine oocyte meiotic maturation.

### 3.4 Depletion of HDAC8 interferes with the recruitment of γ‐tubulin in porcine oocytes

**FIGURE 4:**
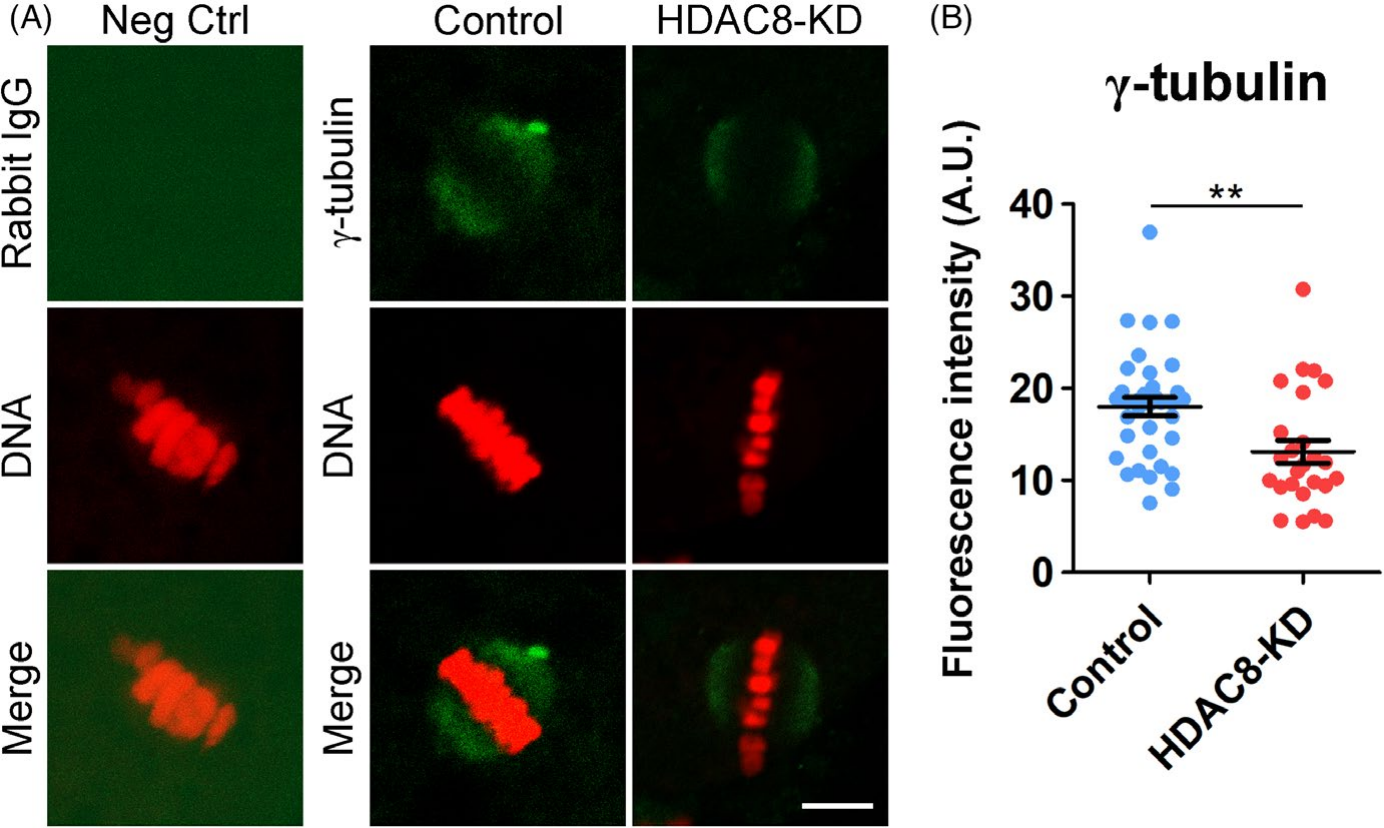
Effect of HDAC8 depletion on the γ‐tubulin dynamics in porcine oocytes. (A) Localization of γ‐tubulin in control and HDAC8‐KD oocytes. M I oocytes were immunostained with anti‐γ‐tubulin antibody and counterstained with PI. Scale bar, 5 μm. (B) The fluorescence intensity of γ‐tubulin signals was measured in control and HDAC8‐KD oocytes. Data were presented as mean value (mean ± SD) of at least three independent experiments. **P < .01

Because spindle assembly requires γ‐tubulin to nucleate the microtubules, we then assessed the influence of HDAC8 depletion on the dynamics of γ‐tubulin. We found that γ‐tubulin was localized at spindle poles with strong signals in M I porcine oocytes (Figure 4A). By contrast, the signals of γ‐tubulin were considerably reduced after HDAC8 depletion, although the localization was not altered (Figure 4A). The quantification of fluorescence signals confirmed the sharp decline of γ‐tubulin at spindle poles following HDAC8 depletion (13.1 ± 1.3, n = 25 vs 18.0 ± 1.0, n = 35, *P* < .01; Figure 4B), implying that HDAC8 is likely to be involved in the recruitment of γ‐tubulin to regulate the spindle assembly in porcine oocytes.

### 3.5 Inhibition of HDAC8 activity disrupts the meiotic maturation of porcine oocytes

**FIGURE 5:**
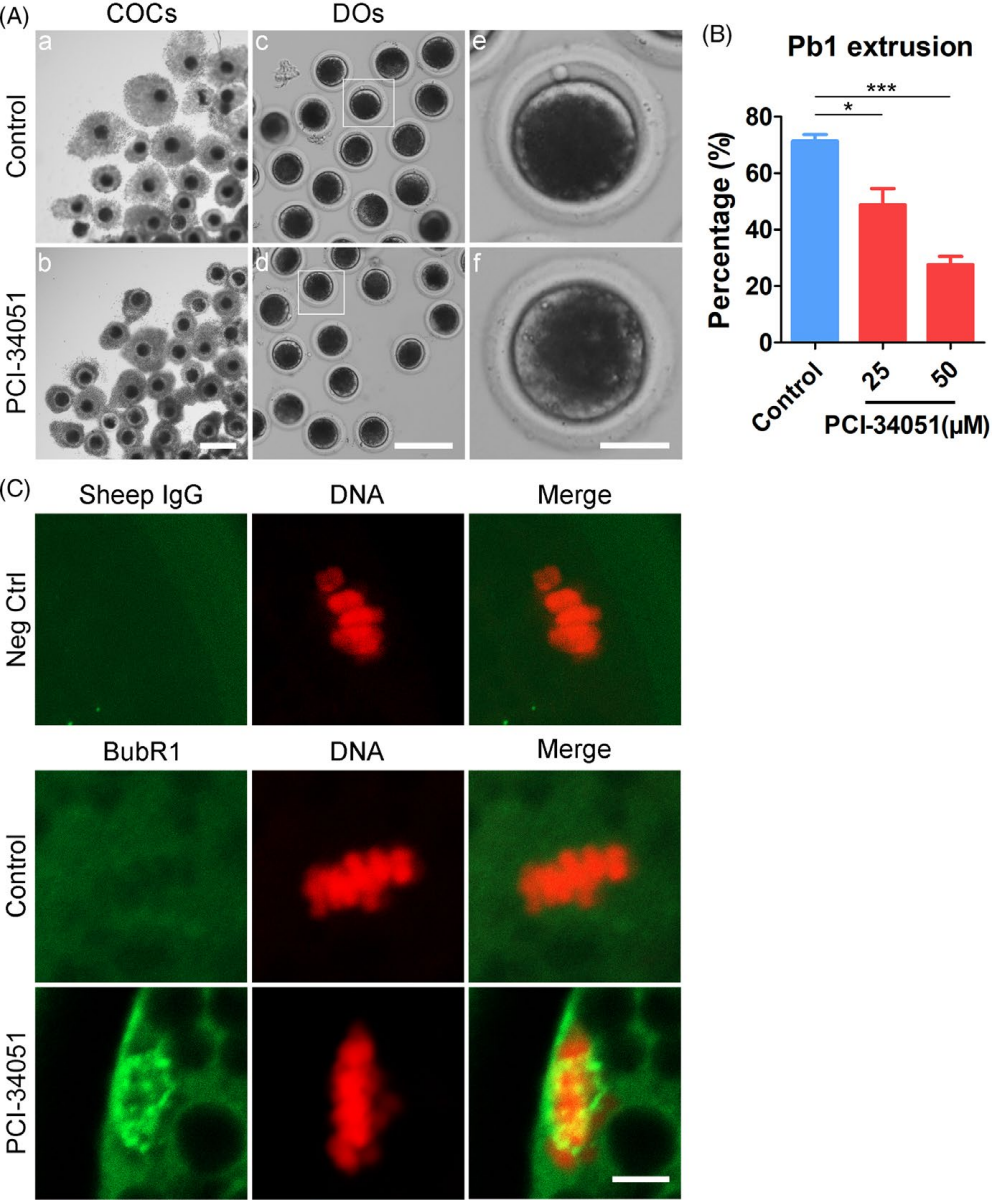
Effect of HDAC8 inhibition on the meiotic progression in porcine oocytes. (A) Representative images of oocytes after in vitro maturation in control and HDAC8‐inhibited (PCI‐34051‐treated) groups. Denuded oocytes (DOs) were collected via removal of cumulus cells from cumulus‐oocyte complexes (COCs). Scale bars, 250 μm (a, b); 200 μm (c, d); 40 μm (e, f). (B) The rate of PBE was quantified in control and HDAC8‐inhibited oocytes. Data were presented as mean percentage (mean ± SEM) of at least three independent experiments. *P < .05, ***P < .001. (C) The localization of BubR1 on the chromosomes in control and HDAC8‐inhibitied oocytes at M I stage. Oocytes were immunostained with anti‐BubR1 antibody and counterstained with PI. Scale bar, 5 μm

To determine whether the function of HDAC8 during porcine oocyte meiosis is dependent on its enzyme activity, we treated oocytes with HDAC8‐specific inhibitor, PCI‐34051, to block its activity. As shown in Figure 5A, treatment of oocytes with different concentrations of PCI‐34051 (25, 50 μM) resulted in failure of oocyte meiotic progression by showing defective cumulus cell expansion and the reduced proportion of PBE after 44 hours of in vitro maturation (control: 71.4 ± 2.3%, n = 98; 25 μmol/L: 48.8 ± 5.8%, n = 102, *P* <.05; 50 μmol/L: 27.6 ± 3.0%, n = 107, *P* <.001; Figure 5B). We used 50 μmol/L PCI‐34051 for subsequent studies because of its more adverse effect on the meiotic maturation. As oocyte meiotic arrest usually results from the activation of spindle assembly checkpoint (SAC), we stained HDAC8‐inhibited oocytes without polar body after in vitro maturation with the antibody to BubR1, a core component of SAC complex. The imaging result showed that BubR1 was unloaded from chromosomes at M I stage in control oocytes, allowing the entry to anaphase stage, whereas BubR1 was still present at the chromosome at M I stage in HDAC‐inhibited oocytes (Figure 5C), indicating that inhibition of HDAC8 activity provokes SAC to arrest the oocyte meiotic maturation.

### 3.6 Inhibition of HDAC8 activity perturbs the spindle/chromosome structure in porcine oocytes

**FIGURE 6:**
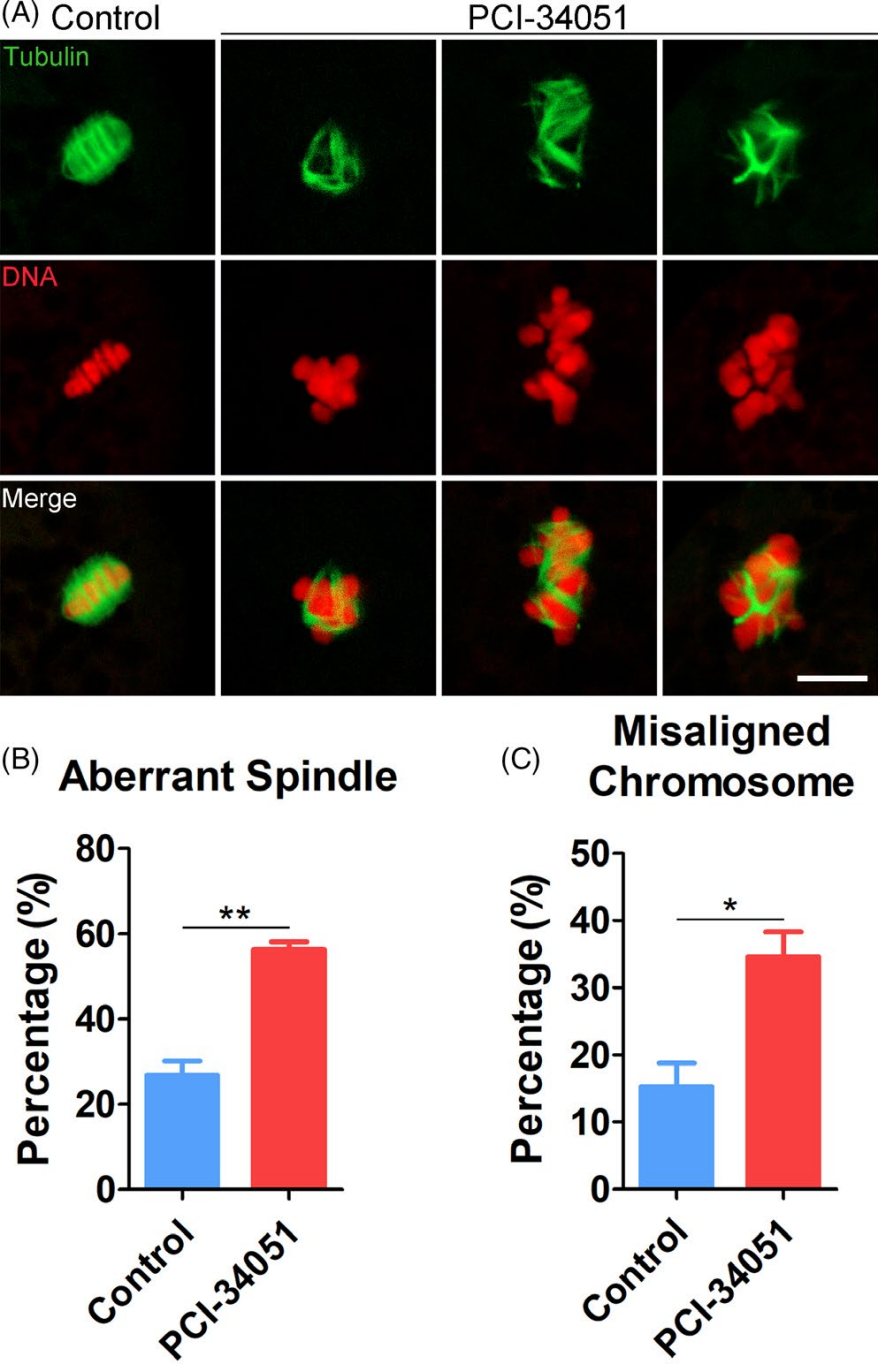
Effect of HDAC8 inhibition on the spindle/chromosome structure in porcine oocytes. (A) Representative images of spindle morphology and chromosome alignment in control and HDAC8‐inhibited oocytes. M I oocytes were immunostained with anti‐α‐tubulin‐FITC antibody and counterstained with PI. Scale bar, 5 μm. (B) The proportion of aberrant spindles was quantified in control and HDAC8‐inhibited oocytes. (C) The proportion of misaligned chromosomes was quantified in control and HDAC8‐inhibited oocytes. Data of (B) and (C) were presented as mean percentage (mean ± SEM) of at least three independent experiments. *P < .05, **P < .01

We next assessed the spindle/chromosome structure in HDAC8‐inhibited oocytes. Spindle morphology and chromosome alignment were visualized by staining oocytes with α‐tubulin‐FITC and PI. As displayed in Figure 6A, the control oocytes formed a standard bipolar spindle apparatus with well‐aligned chromosomes at the equatorial plate. However, a variety of disorganized spindles and misaligned chromosomes was observed in HDAC8‐inhibited oocytes (Figure 6A). Consistently, the quantitative data displayed that a substantially elevated incidence of aberrant spindles (56.4 ± 1.8%, n = 32 vs 26.9 ± 3.3%, n = 35, *P* < .01; Figure 6B) and misaligned chromosomes (34.6 ± 3.8%, n = 32 vs 15.3 ± 3.5%, n = 35, *P* < .05; Figure 6C) was present in HDAC8‐inhibited oocytes, indicating that HDAC8 enzyme activity is essential for the organization of spindle/chromosome structure in porcine oocytes.

### 3.7 Inhibition of HDAC8 activity disturbs the dynamics of γ‐tubulin in porcine oocytes

**FIGURE 7:**
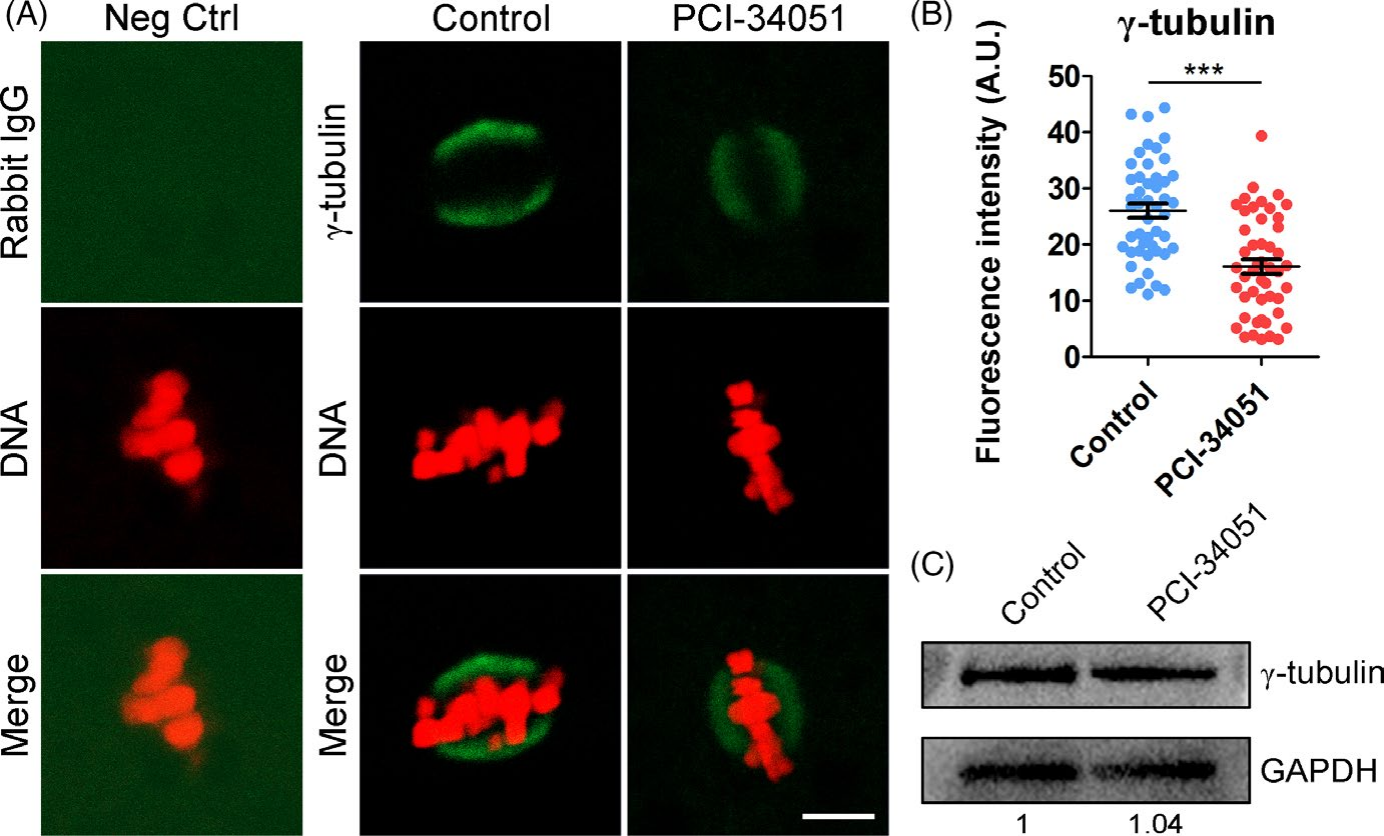
Effect of HDAC8 inhibition on the γ‐tubulin dynamics in porcine oocytes. (A) Localization of γ‐tubulin in control and HDAC8‐inhibited oocytes. M I oocytes were immunostained with anti‐γ‐tubulin antibody and counterstained with PI. Scale bar, 5 μm. (B) The fluorescence intensity of γ‐tubulin signals was measured in control and HDAC8‐inhibited oocytes. Data were presented as mean value (mean ± SD) of at least three independent experiments. ***P < .001. (C) Protein levels of γ‐tubulin in control and HDAC8‐inhibited oocytes. Oocytes in each group were immunoblotted for γ‐tubulin and GAPDH respectively

To ask whether HDAC8 activity is also required for γ‐tubulin dynamics during porcine oocyte meiotic maturation, M I oocytes were stained with γ‐tubulin antibody and then imaged by confocal microscope. The results of immunostaining and fluorescence intensity quantification manifested that inhibition of HDAC8 obviously reduced γ‐tubulin signals at spindle poles compared with controls (16.1 ± 1.3, n = 48 vs 26.0 ± 1.3, n = 48, *P* < .001; Figure 7A,B). Furthermore, we examined the protein level of γ‐tubulin in the oocytes after inhibition of HDAC8. Immunoblotting analysis showed that there was no change in γ‐tubulin protein level in HDAC8‐inhibited oocytes in comparison with the controls (Figure 7C), indicating that loss of HDAC8 activity only affects γ‐tubulin level at spindle poles. Therefore, these results demonstrate that the involvement of HDAC8 in the γ‐tubulin dynamics relies on its enzyme activity.

### 3.8 Inhibition of HDAC8 activity does not change the acetylation level of α‐tubulin in porcine oocytes

**FIGURE 8:**
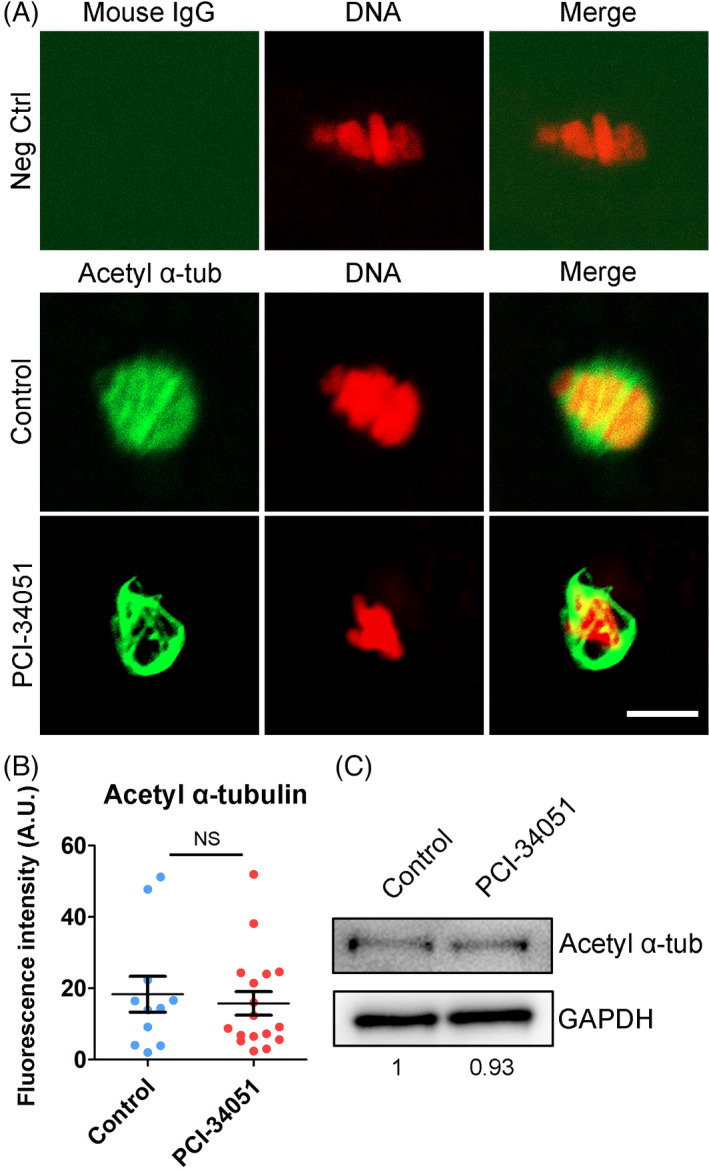
Effect of HDAC8 inhibition on the acetylation level of α‐tubulin in porcine oocytes. (A) Representative images of acetylated α‐tubulin in control and HDAC8‐inhibited oocytes. M I oocytes were immunostained with anti‐acetyl‐α‐tubulin (K40) antibody and counterstained with PI. Scale bar, 5 μm. (B) The fluorescence intensity of acetylated α‐tubulin signals was measured in control and HDAC8‐inhibited oocytes. Data were presented as mean value (mean ± SD) of at least three independent experiments. NS: no significance. (C) Protein levels of acetylated α‐tubulin in control and HDAC8‐inhibited oocytes. Oocytes in each group were immunoblotted for acetylated α‐tubulin and GAPDH respectively

Apart from the microtubule nucleation, microtubule stability is another important factor that affects the spindle assembly. We then evaluated the acetylation level of α‐tubulin, an indicator for the microtubule stability, in HDAC8‐inhibited oocytes. Both immunostaining analysis and fluorescence intensity quantification indicated that HDAC8 inhibition did not cause the significant changes in the level of acetylated α‐tubulin compared with the controls (15.8 ± 3.3%, n = 17 vs 18.4 ± 5.0%, n = 11; Figure 8A,B). This observation was further substantiated by the immunoblotting analysis (Figure 8C). Altogether, our data illustrate that HDAC8 does not play a role in the microtubule stability in porcine oocytes as assessed by the acetylation level of α‐tubulin.

## 4 DISCUSSION

Removal of histone acetyl epigenetic modification by HDACs modulates chromatin structure and transcription, and deacetylation of non‐histones controls diverse cellular processes.^4^, ^12^ Among them, HDAC8 performs critical functions in diverse biological events including mitosis, transcription, chromatin remodelling and RNA splicing and is implicated as a therapeutic target in various diseases including X‐linked intellectual disability, parasitic infections and cancer.^12^, ^27^ In addition, our previous study has shown that HDAC8 participates in the spindle assembly to ensure euploidy in mouse oocytes.^20^ However, whether this function in female germ cells is conserved among species in mammals is unclear. In current study, porcine oocytes were used to address this question.

The predominant cytoplasmic localization of HDAC8 has been reported in both HEK293T and HeLa cells.^28^ Moreover, in HeLa cells, HDAC8 also exhibits nucleolar localization.^28^ Notably, HDAC8 locates at the spindle poles during mouse oocyte meiotic maturation.^20^ Our current findings, however, show a different localization pattern for HDAC8 in porcine oocytes from both mitotic cells and mouse oocytes. Given that the distribution of a molecule in the cells is highly correlated with its function, the spindle localization of HDAC8 in porcine oocytes predicts that it might be implicated in the spindle assembly.

Consistent with our hypothesis, depletion of HDAC8 by RNAi‐mediated silencing approach disrupts spindle/chromosome structure and causes the arrest of the meiotic progression during porcine oocyte maturation by displaying the decreased rate of PBE, a most obvious morphological indicator for the oocyte maturation. As PBE is a complicated cellular event that requires the involvement of many molecules, depletion of HDAC8 did not result in the complete inhibition of PBE. We further validate that these phenotypes induced by HDAC8 depletion result from the impaired recruitment of γ‐tubulin to the spindle poles in porcine oocytes. In animal cells, microtubules are nucleated by the centrosome that is composed of a centriole pair surrounded by pericentriolar material (PCM).^29^ As a key factor in microtubule nucleation, the centrosomal protein γ‐tubulin is first identified as an extragenic suppressor of β‐tubulin mutation in fungus *Aspergillus nidulans* and ubiquitously exists in eukaryotic cells.^30^, ^31^ Studies have shown that γ‐tubulin is present in every major microtubule‐organizing centre (MTOC), including the spindle pole bodies of *Schizosaccharomyces pombe* and *A nidulans*,^32^, ^33^ as well as the centrosomes of *Drosophila, Xenopus* and mammalian cells.^34^, ^35^ γ‐tubulin associates with other proteins to form multiprotein γ‐tubulin ring complexes (γ‐TuRCs) that template and catalyse the assembly of microtubules.^36^ However, how HDAC8 controls the γ‐tubulin dynamics in oocytes needs further investigations.

Another finding in our study is that the role of HDAC8 in the porcine oocyte meiosis is dependent on its enzyme activity. Inhibition of HDAC8 activity using its specific inhibitor PCI‐34051 photocopies the phenotypes of HDAC8 depletion by RNAi, including meiotic progression, spindle assembly and γ‐tubulin dynamics. In addition, we document that the arrest of oocyte maturation caused by inhibition of HDAC8 results from SAC activation.

Acetylation of α‐tubulin at the N‐terminal lysine 40 (K40) serves as a marker for the presence of stable microtubules, which affects the activity of microtubule‐associated proteins and microtubule‐based motors.^37^, ^38^ In both HeLa and HEK293 cells, HDAC8 interacts with α‐tubulin and deacetylates it to control its functionality.^28^ Our data reveal that HDAC8 is not essential for deacetylation of α‐tubulin in porcine oocytes although it is involved in the spindle assembly, indicating that HDAC8 inhibition‐caused spindle defects are not due to the impairment of microtubule stability. Collectively, in mitotic and meiotic cells, HDAC8 participates in the spindle formation through different mechanisms, and its role in female germ cells is highly conserved between mice and pigs.

In summary, we document that HDAC8 locates to the spindle apparatus in porcine oocytes to take part in the spindle assembly via recruitment of γ‐tubulin, and hence driving the oocyte meiotic maturation. This function of HDAC8 relies on its enzyme activity and is conserved among species in mammals.

## CONFLICT OF INTEREST

The authors declare that they have no conflict of interest.

## AUTHOR CONTRIBUTIONS

BX designed the research. YC, CP, YL and YM performed the experiments. YC and BX analysed the data. YC, YM and BX wrote the manuscript.

## Data Availability

The data used to support the findings of this study are available from the corresponding author upon request.
